# The effects of chiropractic adjustment on inattention, hyperactivity, and impulsivity in children with attention deficit hyperactivity disorder: a pilot RCT

**DOI:** 10.3389/fpsyg.2024.1323397

**Published:** 2024-05-06

**Authors:** Imran Amjad, Imran Khan Niazi, Nitika Kumari, Jens Duehr, Gulyana Shehzad, Usman Rashid, Jenna Duehr, Robert J. Trager, Kelly Holt, Heidi Haavik

**Affiliations:** ^1^Centre for Chiropractic Research, New Zealand College of Chiropractic, Auckland, New Zealand; ^2^Physical Therapy Department, Riphah International University, Islamabad, Pakistan; ^3^Faculty of Health & Environmental Sciences, Health & Rehabilitation Research Institute, AUT University, Auckland, New Zealand; ^4^Department of Health Science and Technology, Aalborg University, Aalborg, Denmark; ^5^National Intitute of Psychology, Quid e Azam University, Islamabad, Pakistan; ^6^Connor Whole Health, University Hospitals Cleveland Medical Center, Cleveland, OH, United States

**Keywords:** ADHD (attention deficit and hyperactivity disorder), chiropractic adjustment, Vanderbilt ADHD Diagnostic Teacher Rating Scale, Swanson, Nolan and Pelham Teacher and Parents Rating Scale, pilot RCT, ADHD Rating Scale-IV

## Abstract

**Background:**

Attention deficit hyperactivity disorder (ADHD) is a neurobiological disorder characterized by inattention, hyperactivity, and impulsivity. We hypothesized that chiropractic adjustments could improve these symptoms by enhancing prefrontal cortex function. This pilot study aimed to explore the feasibility and efficacy of 4 weeks of chiropractic adjustment on inattention, hyperactivity, and impulsivity in children with ADHD.

**Methods:**

67 children with ADHD were randomly allocated to receive either chiropractic adjustments plus usual care (Chiro+UC) or sham chiropractic plus usual care (Sham+UC). The Vanderbilt ADHD Diagnostic Teacher Rating Scale (VADTRS), Swanson, Nolan and Pelham Teacher and Parents Rating Scale (SNAP-IV), and ADHD Rating Scale-IV were used to assess outcomes at baseline, 4 weeks, and 8 weeks. Feasibility measures such as recruitment, retention, blinding, safety, and adherence were recorded. Linear mixed regression models were used for data analysis.

**Results:**

56 participants (mean age ± SD: 10.70 ± 3.93 years) were included in the analysis. Both the Chiro+UC and Sham+UC groups showed significant improvements in total and subscale ADHD scores at 4 weeks and 8 weeks. However, there were no significant differences between the two groups.

**Conclusion:**

This pilot study demonstrated that it was feasible to examine the effects of chiropractic adjustment when added to usual care on ADHD outcomes in children. While both groups showed improvements, the lack of significant between-group differences requires caution in interpretation due to the small sample size. Further research with larger samples and longer follow-up periods is needed to conclusively evaluate the effects of chiropractic adjustments on ADHD in children.

## 1 Introduction

Attention deficit hyperactivity disorder (ADHD) is a neurodevelopmental disorder characterised by hyperactivity, impulsivity, and inattention (Sharma and Couture, 2014). Among individuals up to age 18 years, ADHD has a global prevalence of 7 % (Thomas et al., 2015). There are three subtypes of ADHD: predominantly hyperactive–impulsive, predominantly inattentive, and a combined type of the first two (American Psychiatric Association, 2013; Sharma and Couture, 2014). While medications and behavioral therapies are the most effective and most common treatments for ADHD, parents frequently seek complementary and alternative therapies such as chiropractic care to address associated symptoms or comorbid conditions.

Several hypotheses for the neurobiological basis of ADHD have been proposed. Earlier studies observed a reduced function and volume of the white and grey matter of the brain associated with impaired planning, cognition, attention and behavior (Cortese, 2012). Recent studies have proposed the involvement of the prefrontal cortex in the development of ADHD, as this area of the brain is mainly involved in the regulation of attention and behavior (Arnsten and Pliszka, 2011; Kesner and Churchwell, 2011; Mehta et al., 2019). Studies have shown a slower maturation, reduced activity, and reduced volume of the prefrontal cortex in patients with ADHD (Shaw et al., 2007; Arnsten and Pliszka, 2011). The slower maturation of the prefrontal cortex is thought to be associated with the continuation of ADHD into adulthood (Shaw et al., 2011).

Several research studies have also identified neurological deficits in somatosensory processing and sensorimotor integration in both children and adults with ADHD (Ghanizadeh, 2011). These deficits have been observed using neuroimaging techniques, such as fMRI, where elevated resting-state activity in basic sensory and sensory-related cortices were found in adolescents with ADHD compared to matched controls (Tian et al., 2008). Additionally, other studies have identified dysfunctional neural activity in somatosensory cortices and evidence of reduced sensorimotor responses (Dockstader et al., 2008; Frost-Karlsson et al., 2024). Functionally, impaired sensorimotor processing is well documented in the literature, with ADHD children displaying sensory hypersensitivity and difficulty in filtering out intrusive sensory or motor stimuli (Lane and Reynolds, 2019).

Individuals with ADHD are affected by a functional impairment of behavioral, academic and social functioning (DuPaul et al., 2001; Barbaresi et al., 2007; Solanto et al., 2009), and demonstrate a deficit in executive functions (e.g., memory, executive attention, planning, task switching, and response inhibition) across multiple neuropsychological tasks (Marije Boonstra et al., 2005; Martel et al., 2007). Children with ADHD often display impaired attention development, slower processing of information, hyperactivity, and executive function deficits leading to poor performance on standardised tests, lower grades, and increased likelihood of dropping out of school (Gillberg, 1998; Childress and Berry, 2012). Additionally, ADHD presents with one or more comorbidities such as mood disorders, anxiety disorders, oppositional defiant and conduct disorders (Gillberg, 1998).

Treatment of ADHD children commonly involves a multidisciplinary approach, including pharmacotherapy and/or behavioral modification (Sharma and Couture, 2014). In a study using data from 2011 to 2014 from the United States, the most common therapy used at any point by children with ADHD was medication (90%), while 62% of children had used at least one form of psychosocial or behavioral therapy (Danielson et al., 2018). While evidence supports short-term efficacy of pharmacological treatments, there are certain drawbacks, such as limited evidence for long-term efficacy, as well as potential safety concerns (Brams et al., 2008; Lerner and Wigal, 2008; Caye et al., 2019). Accordingly, parents of children with ADHD often seek out non-pharmacological treatments such as forms of complementary and alternative medicine (Xue et al., 2021).

A survey study using United States data from 2012 and 2017 found that 19% of children with ADHD used complementary and alternative medicine for their symptoms. After meditation (11%), yoga (10%), and breathing exercises (7%), chiropractic (3%) was the fourth most commonly used of these therapies (Wang et al., 2020). Chiropractic is a healthcare profession that most often manages neuromusculoskeletal conditions (Beliveau et al., 2017). Chiropractors frequently treat these conditions using high velocity low amplitude thrust (i.e., adjustment), a manual treatment directed to dysfunctional joints of the spine.

There is little evidence to support the efficacy of chiropractic interventions for ADHD. Two reviews on the topic, published in 2010, reported that there was limited research to suggest that chiropractic adjustment was beneficial for individuals with ADHD (Ferrance and Miller, 2010; Karpouzis et al., 2010). Since these reviews were published, one case series (*n* = 4), and multiple case reports described improvements in ADHD potentially related to chiropractic adjustments (Wittman et al., 2009; Alcantara and Davis, 2010; Muir, 2012; Fairest et al., 2019). A pilot and feasibility randomized controlled crossover trial (*n* = 30) found that a single session of chiropractic adjustment led to a significant improvement in reading time measured by an eye tracker in children with ADHD (Cade et al., 2021). While reading time can provide insights into certain aspects of attention and eye movements related to reading, it may not capture the full range of ADHD symptoms or assess other domains of impairment. Due to these limited, but insufficient findings, previous authors have proposed that the effect of chiropractic adjustment on clinical outcomes be investigated for individuals with ADHD using randomized controlled trials (Lystad and Pollard, 2009; Karpouzis et al., 2010; Cade et al., 2021). Finally, a survey of chiropractic research priorities in Australia highlighted ADHD as an often-requested topic among practitioners (Amorin-Woods et al., 2023).

However, some information may be gleaned from the osteopathic literature considering osteopaths use spinal manipulative interventions potentially like chiropractic adjustments. In one small randomized controlled trial (*n* = 28), children with ADHD receiving osteopathic manipulative therapy were found to have significant improvement in Biancardi-Stroppa Test scores (a measure of visual–spatial attention) compared to those receiving conventional care only (Accorsi et al., 2014). However, as reinforced by a recent review article, additional available research on the topic is limited (Posadzki et al., 2022).

Several studies have shown that chiropractic adjustments alter somatosensory processing, sensorimotor integration, and motor control in people with subclinical spinal pain, healthy individuals with evidence of spinal dysfunction and people with chronic stroke (Marshall and Murphy, 2006; Taylor and Murphy, 2007a,b, 2008, 2010a,b; Haavik and Murphy, 2011; Haavik and Murphy, 2012; Niazi et al., 2015; Holt et al., 2019; Navid et al., 2019). This suggests that chiropractic adjustments have a neural plastic effect on the central nervous system (CNS) (Haavik and Murphy, 2012; Pickar and Bolton, 2012; Niazi et al., 2015), and in particular, the prefrontal cortex (Lelic et al., 2016). People with ADHD have been identified as having dysfunctional somatosensory processing and sensorimotor integration and deficits in executive functions and this is thought to be related to many of the typical ADHD symptoms (DuPaul et al., 2001; Marije Boonstra et al., 2005; Martel et al., 2007; Dockstader et al., 2008; Tian et al., 2008; Ghanizadeh, 2011; Frost-Karlsson et al., 2024). Given the previous literature that has shown changes following chiropractic adjustments to these regions and processes of the brain typically implicated in ADHD, we reasoned that it would be a logical next step to investigate whether chiropractic adjustments could be beneficial for people with ADHD.

The American Academy of Child and Adolescent Psychiatry recommends utilising outcome measures for attention, hyperactivity and impulsive symptoms of ADHD because patients with ADHD are referred for treatment due to the presence of functional impairments (Pelham et al., 2005; Pliszka and AACAP Work Group on Quality Issues, 2007; Epstein and Weiss, 2012). The Academy further suggests incorporating behavior rating scales in the assessment of ADHD treatment response (Pelham et al., 2005; Pliszka and AACAP Work Group on Quality Issues, 2007; Karpouzis et al., 2010; Epstein and Weiss, 2012).

Given the limited and predominantly observational research on the topic of ADHD and chiropractic adjustments, we sought to conduct a randomized controlled pilot study to assess the feasibility, and safety and objectively examine the effect of this therapy on validated outcome measures for ADHD symptoms. We hypothesized that there would be a significant improvement in the ADHD scales after 4 weeks of chiropractic adjustments in children with ADHD.

## 2 Methods

### 2.1 Design and setting

This was a parallel-group, pilot, randomized controlled trial (RCT). The study was conducted at the *Army Special Education School and Rehabilitation Center for Special and Slow Learner Children, Rawalpindi, Pakistan*, from January to June 2019. The Ethical Review Committee of Riphah International University, Pakistan, approved the study (Riphah/RCRS/REC/000459). In addition, the study was registered with the National Institutes of Health ClinicalTrials.gov clinical trial registry (NCT03849807).

### 2.2 Study participants

Participants were recruited from the *Army Special Education School and Rehabilitation Center for Special and Slow Learner Children*. This study refers to these participants as having ADHD, based on a previous clinical diagnosis. Participants from age five to 17 years, diagnosed with ADHD by a pediatrician at the specialist center of Army Special Education School and Rehabilitation Center for Special and Slow Learner Children, and whose parents agreed to their participation, were included in the study.

Participants were excluded if they showed no evidence of spinal dysfunction (i.e., presence of chiropractic subluxation indicators identified by a chiropractor), had absolute contraindications to chiropractic adjustments (i.e., spinal fracture, atlantoaxial instability, spinal infection, spinal tumor, or cauda equina syndrome), or previously had a serious adverse event related to chiropractic adjustment(s).

All children were recruited from schools after obtaining written consent from their parents and the school administration. There is limited knowledge of chiropractic in this region, as this is an infrequently used treatment. An explanation session (with administration and teachers) was given by a researcher about the research project and chiropractic care at the time of ethical approval from the school’s administration.

### 2.3 Procedure

Following recruitment and screening, participants were randomly allocated to either 4 weeks of chiropractic adjustment plus usual care (Chiro+UC) or 4 weeks of sham chiropractic adjustment plus usual care (Sham+UC). Randomization was carried out following the baseline assessment using an online minimization tool (QMinim, Telethon Kids Institute, Australia) (Saghaei and Saghaei, 2011). All baseline demographic data and medical history were taken from the school record and teachers. Age and gender were used as input for minimization. All participants, the outcomes assessors (psychologist and teachers), and the teachers providing the usual intervention were blinded to group allocation. The statistician who analyzed the data was also blinded to group allocation, as all recorded data were anonymized and coded before being provided for analysis. The chiropractors providing chiropractic adjustment could not be blinded to group allocation. The primary outcome measures were assessed at baseline, after 4 weeks of intervention (post-intervention), and at eight-week follow-up, i.e., 4 weeks after the four-week intervention (to assess retention effects).

### 2.4 Interventions

The interventions were 4 weeks of chiropractic adjustments plus usual care (Chiro+UC) and 4 weeks of sham chiropractic adjustments plus usual care (Sham+UC). A standalone chiropractic intervention was not considered in this pilot study, as this would have meant withholding an intervention (i.e., usual care) that is known to be effective to test a novel intervention (i.e., chiropractic adjustments).

#### 2.4.1 Chiropractic

**Figure 1:**
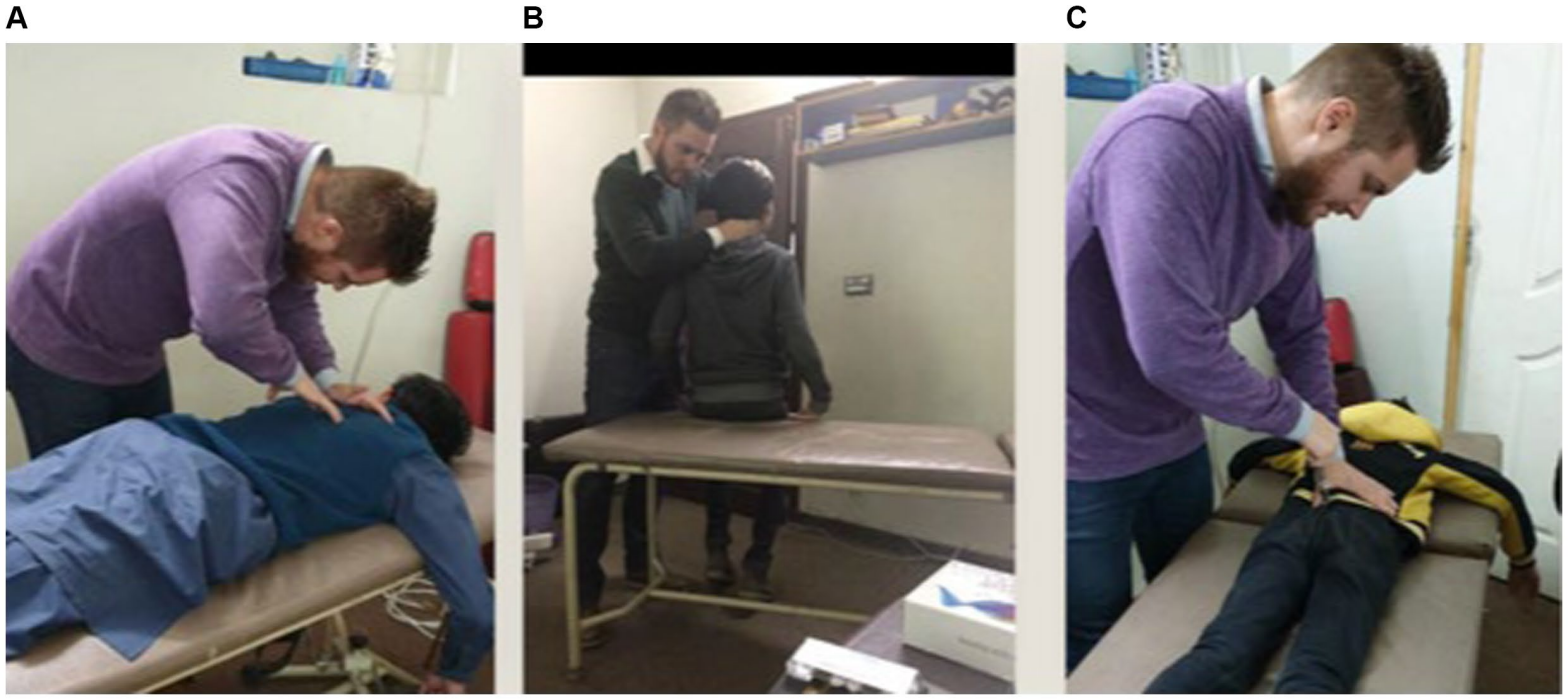
Chiropractor delivers high-velocity, low-amplitude thrusts manually **(A,B)** or via the Activator **(C)** at vertebral segments deemed appropriate based on the chiropractic examination.

In the chiro+UC group, New Zealand registered chiropractors checked participants for spinal dysfunction/subluxation and performed chiropractic adjustments at these spinal levels, where necessary, during the intervention period. Participants were examined and treated by the chiropractor approximately three times per week for 4 weeks. Clinical indicators for spinal dysfunction/subluxation included tenderness to palpation, restricted intersegmental motion, asymmetric muscle tension and blocked joint-play or end-feel. These clinical indicators are routinely used by chiropractors when analyzing the spine and have previously been shown to be reliable for identifying spinal dysfunction/subluxation when used within a multidimensional battery of tests (Triano et al., 2013; Holt et al., 2018). Chiropractic adjustments were individualized to each participant based on their clinical findings and provided where clinically warranted using either manual, high-velocity, low-amplitude thrust or instrument-assisted thrust to the spine or pelvic joints (Cooperstein and Gleberzon, 2004). Instrument-assisted adjustments were performed using an Activator instrument, which is a hand-held device that delivers a high-velocity, low-amplitude thrust. This thrust can be set at various pre-determined force levels and directed at dysfunctional spine or pelvic joints. Multiple levels of the spine were adjusted in each participant if deemed appropriate based on the chiropractic examination (Figure 1). Each chiropractic visit lasted approximately 15 min. The chiropractor provided no other interventions.

#### 2.4.2 Sham chiropractic adjustments

To reduce the impact of contextual effects on study outcomes, the control group received sham chiropractic adjustment along with usual care. Participants in the Sham+UC group saw the same chiropractor at the same frequency, as those in the experimental group. The chiropractor performed the same assessment for spinal dysfunction as the experimental group and chiropractic visits were roughly the same as those in the experimental group. However, instead of applying manual or instrument-assisted thrusts to the spine, the chiropractor either (1) positioned participants as if they were going to thrust on the spine, but did not provide a manual thrust, or (2) they placed an adjusting instrument, set to the minimum setting, lateral to the spine or on the chiropractor’s hand or arm and produced a clicking sound with the instrument. Translators were used to facilitate communication between the chiropractors and participants, for example when asking participants to move into the required positions for the control and experimental procedures.

#### 2.4.3 Usual care

The participants were already on certain plans of care according to their individual needs in school. The school provided their own trained staff for all relevant care and services, apart from medications. The usual care provided by the school and affiliated staff included psychological rehabilitation (Braswell and Bloomquist, 1991), speech therapy (Barona-Lleo and Fernandez, 2016), physical therapy (Fliers et al., 2010; Kumari et al., 2020), occupational therapy, medications (Swanson et al., 1995) and cognitive therapies by teachers and family (Braswell and Bloomquist, 1991; Pelham et al., 2000). All these care plans were going in parallel with the study plan. Considering these interventions standard-of-care for children with ADHD, and included participants were already receiving this care, it was ethically necessary to have both groups continue to receive these therapies. There was no spinal manual therapy or chiropractic adjustment provided in the usual care group.

### 2.5 Outcome measures

All outcome measures were based on questionnaires which were written in English. An expert psychologist who was bilingual in English and Urdu helped the teachers and parents complete each outcome measure. All involved teachers were also bilingual, which further facilitated this step. Baseline readings were collected after recruitment and then the first visit was planned for each participant.

The primary outcome measures included the Vanderbilt ADHD Diagnostic Teacher Rating Scale (VADTRS), Swanson, Nolan and Pelham Teacher and Parents Rating Scale (SNAP-IV), and ADHD Rating Scale-IV (Epstein and Weiss, 2012). The VADTRS is a 43-item scale primarily focused on ADHD symptoms which are each scored using a four-or five-point Likert scale (Collett et al., 2003). The VADTRS items assess impairment in areas such as reading, writing, mathematics, and relationships. This scale measures behavior problems at school in four subscales: Inattention, Hyperactivity/Impulsivity, Oppositional Defiant/Conduct Disorder, and Anxiety/Depression, wherein greater scores are associated with more problems (Wolraich et al., 1998). The VADTRS demonstrates good to excellent concurrent validity and internal consistency (Collett et al., 2003).

The SNAP-IV is a 90-item symptom scale that includes the ADHD subscales of Inattention, Hyperactivity/Impulsivity, and Opposition/Defiance along with summary questions in each domain, with each item measured using a four-point Likert scale (Swanson, 1992). The SNAP-IV displays good to excellent internal consistency (Stevens and Quittner, 1998; Collett et al., 2003), and has been validated for use as an outcome measure with randomized controlled trials in the ADHD (Hall et al., 2020). In this outcome measure, higher scale ratings correlate with a diagnosis of ADHD (Hall et al., 2020).

The ADHD Rating Scale-IV is an 18-item checklist that measures symptoms of ADHD according to diagnostic criteria within the Diagnostic and Statistical Manual of Mental Disorders, with items rated via a four-point Likert scale (Pappas, 2006). While there are two versions of this scale available (Pappas, 2006), the current study used the home version. The ADHD Rating Scale-IV includes two expected subscales: (1) Inattentive and (2) Hyperactive/ Impulsive (DuPaul et al., 1997, 1998a,b). This scale has good to excellent internal consistency and test–retest reliability (Collett et al., 2003). Higher scores are positively correlated with markers of ADHD and negatively correlated with accuracy on academic tasks (Pappas, 2006).

### 2.6 Feasibility parameters

Trial feasibility was assessed by (1) recruitment rate, (2) retention and follow-up rates, (3) queries from the parents and teachers, (4) data collection, (5) feasibility and time required for the data collection from teachers and parents, (6) randomization and blinding, (7) understanding the questions and other data collection methods by parents and teachers and (8) reporting of any possible adverse events or complications. The recruitment rate was measured by recording the number of participants considered, screened and included, while the retention and follow-up rate was measured by recording the number of drop outs and reasons for drop out. Potential adverse events were determined by asking the physical therapists, teacher and translators assisting the chiropractors to ask participants, at scheduled intervention visits, about any injuries or perceived adverse effects of care that may have occurred during the trial. At the end of the study, teachers were asked whether they had observed any changes in the childrens’ behaviors and activities.

### 2.7 Statistical analysis

Statistical analysis was conducted to evaluate the mean change scores for the outcomes. The change scores were obtained by subtracting the post-experiment scores from pre-experiment scores. In this way, a positive change-score represented a decrease in outcome score from the pre-experiment time point to the post-experiment time point. A multivariate longitudinal analysis of covariance model was used to estimate post-experiment mean change-scores across groups while adjusting for baseline scores. The analysis employed a linear mixed regression model. In addition to a full interaction between time, group and outcome; the model also included correlated participant-wise random intercepts at each time-point (post and follow-up). These random intercepts allowed the model to account for correlations arising from repeated measurements. This random effects structure was chosen from amongst a number of other possible structures by minimising Akaike’s Information Criterion adjusted for small samples. The model goodness-of-fit was evaluated by inspecting the normality and homogeneity of variance of its residuals using QQ-plot, fitted-values versus residuals plot and a histogram. The post-intervention mean change-scores for the groups and differences in change-scores across the groups were reported along with their 95% confidence intervals. The statistical significance of the mean change-scores and differences across groups was evaluated with Z-and T-tests. These tests were based on the model estimates for mean change scores, their standard error and the accompanying degrees of freedom. The statistical significance level was set at 0.05. The analysis was conducted in R (R Foundation for Statistical Computing) version 4.1.0 using packages lme4 version 1.1–27, emmeans version 1.6.1 and dplyr version 1.0.6 (RR Core Team, 2013; Bates et al., 2015; Wickham et al., 2015; Lenth et al., 2016). The access date for these packages was June 28, 2021.

We utilized a convenience sampling approach rather than a formal statistical calculation for sample size. We recruited potential participants during a pre-defined two-week window, considering our limited access to the study environment and resources, which prioritized practicality and allowed for sufficient follow-up time to gather data.

## 3 Results

### 3.1 Trial feasibility

#### 3.1.1 Recruitment rate

**Figure 2:**
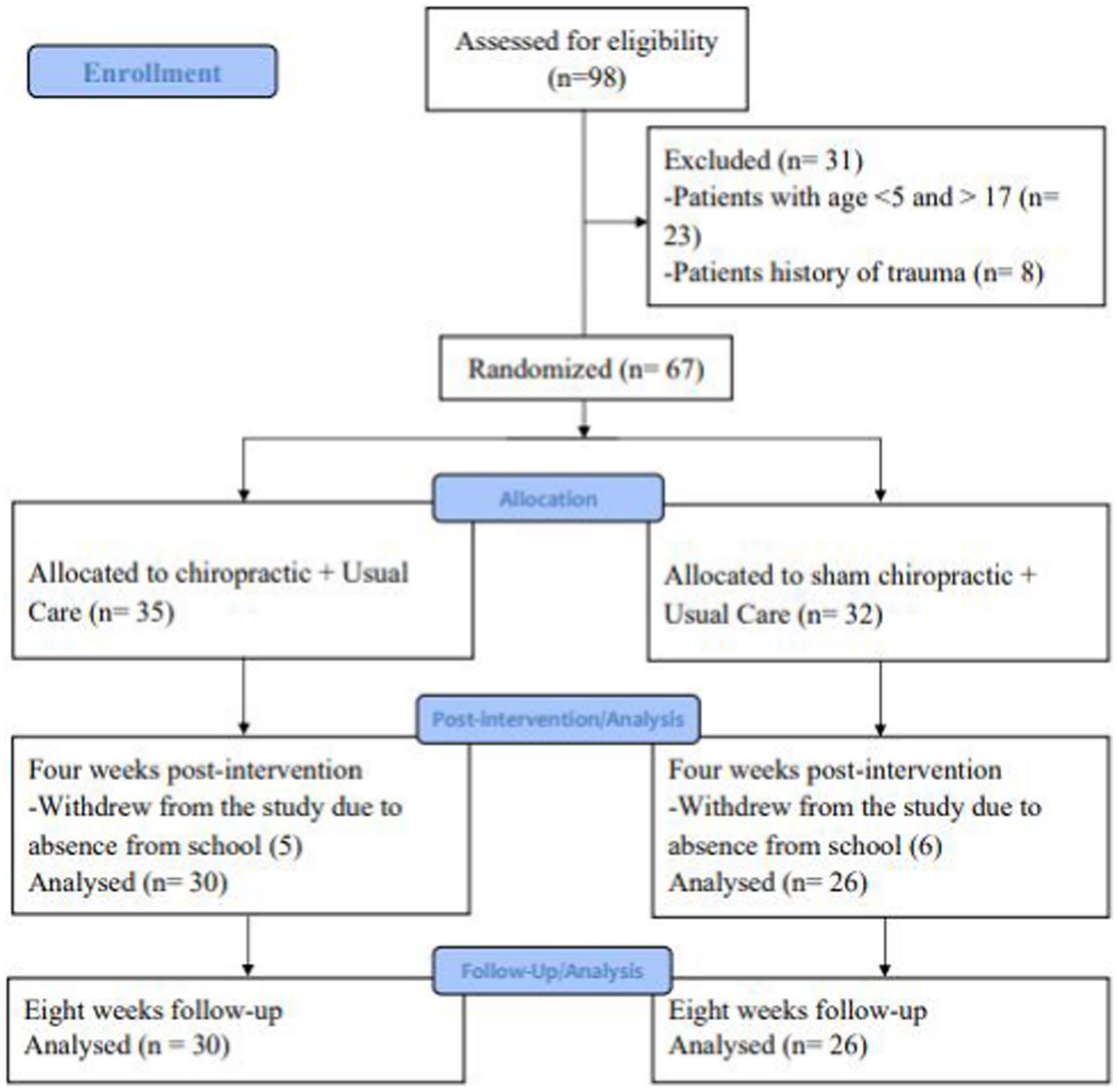
CONSORT study flow diagram.

Participant recruitment was done for 2 weeks. During this recruitment period ninety-eight participants were screened for eligibility, of which 31 were deemed ineligible as they were aged less than five or greater than 17 years or had a history of trauma. The remaining 67 participants were enrolled in the study (Figure 2) (see Table 1).

**Table 1:** Baseline characteristics of participants in each group.

Variables	Chiro+UC	Sham+UC
Gender		
Male (*n*)	22 (73%)	19 (73%)
Female (*n*)	8 (27%)	7 (27%)
Age, years (mean +/− SD)	11.33 ± 4.44	9.96 ± 3.15

chiro, chiropractic adjustment; n, number of participants; SD, standard deviation; UC, usual care.

#### 3.1.2 Retention and follow-up rate

Fifty-six (84%) participants completed the post-intervention assessment 26 from the Sham+UC group and 30 from the Chiro+UC group (mean age ± SD: 10.70 ± 3.93 years). A total of 11 children withdrew due to absence from school during the study period. There were no dropouts during the follow-up assessment window.

#### 3.1.3 Queries and concerns of parents and teachers

There were a few queries and questions about the method and evidence of chiropractic care at the time of ethical approval from the school’s administration. No direct queries were asked by the parents from the researchers.

#### 3.1.4 Data collection

We encountered some challenges during the data collection process, including difficulty in recruiting participants due to limitations in accessing the classes due to the school operating hours. The outcome measures were completed by parents and four class teachers responsible for the involved classes. The average time taken to complete the survey questionnaire was 30 min. Feedback from teachers indicated that the tools were easy to understand and complete.

#### 3.1.5 Blinding

Regardless of group allocation, more than 95% of the teachers of the participants in the present study believed that the children had undergone an active chiropractic intervention, which indicates adequate blinding of the participants. This is difficult to achieve in trials involving a manual intervention (Chaibi et al., 2015) and is a strength of the present study.

#### 3.1.6 Qualitative feedback

A qualitative descriptive content analysis of participants’ responses revealed two key themes. Teachers tended to note improvements in the behavior of the children and found no signs or symptoms of any adverse event.

#### 3.1.7 Adverse events

There were no reported adverse events in both groups throughout the course of the study.

### 3.2 Outcome measures

#### 3.2.1 VADTRS scale

The within-group analysis of the VADTRS scale showed that there was significant change in total score, Aggression/Defiance, Hyperactivity and Impulsivity, Inattention, and Opposition/Defiance subscales in both the Chiro+UC group and the Sham+UC group at four-week post-intervention and eight-week follow-up (Table 2). The between-group analysis of the VADTRS scale showed that there was no significant difference in the total score, Aggression/Defiance, Hyperactivity and Impulsivity, Inattention, and Oppositional defiant disorder between the Chiro+UC group and Sham+UC group at four-week post-intervention and eight-week follow-up (Table 3). The clinical important difference VADTRS scale is six points.

**Table 2:** Within-group change scores for the Vanderbilt ADHD Diagnostic Teacher Rating Scale (VADTRS) (pre-minus post-experiment raw score) across groups and time points for various subscales.

Outcome	Group	Time	Estimate ± SE [95% CI]	H_0_: Estimate = 0 t [df], *p*
Total Score	Chiro+UC	Post	9 ± 1[6, 12]	6.397 [92.4], <0.001
	Sham+UC	Post	9 ± 2[6, 12]	5.636 [92.4], <0.001
	Chiro+UC	Follow-up	9 ± 1[6, 12]	6.235 [92.4], <0.001
	Sham+UC	Follow-up	10 ± 2[7, 13]	6.382 [92.4], <0.001
A/D	Chiro+UC	Post	3.16 ± 0.44 [2.29, 4.02]	7.214 [135], <0.001
	Sham+UC	Post	3.09 ± 0.46 [2.19, 4]	6.758 [132], <0.001
	Chiro+UC	Follow-up	2.71 ± 0.48 [1.76, 3.66]	5.648 [108], <0.001
	Sham+UC	Follow-up	2.89 ± 0.53 [1.84, 3.93]	5.475 [112], <0.001
HI	Chiro+UC	Post	1.63 ± 0.43 [0.78, 2.48]	3.783 [130], <0.001
	Sham+UC	Post	1.13 ± 0.45 [0.24, 2.03]	2.504 [128], 0.014
	Chiro+UC	Follow-up	1.73 ± 0.47 [0.79, 2.67]	3.661 [104], <0.001
	Sham+UC	Follow-up	0.97 ± 0.52 [−0.07, 2.01]	1.853 [108], 0.067
IA	Chiro+UC	Post	1.53 ± 0.43 [0.68, 2.39]	3.556 [130], 0.001
	Sham+UC	Post	1.17 ± 0.45 [0.27, 2.06]	2.566 [129], 0.011
	Chiro+UC	Follow-up	2.53 ± 0.47 [1.59, 3.47]	5.348 [104], <0.001
	Sham+UC	Follow-up	1.66 ± 0.52 [0.62, 2.7]	3.17 [109], 0.002
ODD	Chiro+UC	Post	2.75 ± 0.43 [1.9, 3.59]	6.429 [127], <0.001
	Sham+UC	Post	3.3 ± 0.45 [2.41, 4.19]	7.321 [127], <0.001
	Chiro+UC	Follow-up	2.33 ± 0.47 [1.4, 3.26]	4.961 [102], <0.001
	Sham+UC	Follow-up	2.64 ± 0.52 [1.6, 3.68]	5.051 [108], <0.001

These results show a significant reduction in each subscale score for both Chiro+UC and Sham+UC groups. A/D, Aggression/Defiance; CI, confidence intervals; HI, Hyperactivity and Impulsivity; IA, Inattention; ODD, Oppositional defiant disorder; SE, standard error.

**Table 3:** Between-group differences in change scores for various outcomes at post-experiment and follow-up time points for the Vanderbilt ADHD Diagnostic Teacher Rating Scale (VADTRS).

Outcome	Contrast	Time	Difference ± SE [95% CI]	H_0_: Difference = 0 t [df], *p*
Total Score	Chiro+UC – Sham+UC	Post	0 ± 2 [−4, 5]	0.234 [92.3], 0.816
	Chiro+UC – Sham+UC	Follow-up	-1 ± 2 [−5, 3]	0.423 [92.3], 0.674
A/D	Chiro+UC – Sham+UC	Post	0.07 ± 0.62 [−1.16, 1.29]	0.106 [127], 0.916
	Chiro+UC – Sham+UC	Follow-up	−0.18 ± 0.7 [−1.57, 1.21]	−0.256 [105], 0.798
HI	Chiro+UC – Sham+UC	Post	0.5 ± 0.62 [−0.73, 1.72]	0.8 [127], 0.425
	Chiro+UC – Sham+UC	Follow-up	0.76 ± 0.7 [−0.63, 2.16]	1.086 [105], 0.28
IA	Chiro+UC – Sham+UC	Post	0.37 ± 0.62 [−0.86, 1.6]	0.592 [127], 0.555
	Chiro+UC – Sham+UC	Follow-up	0.87 ± 0.7 [−0.52, 2.26]	1.239 [105], 0.218
ODD	Chiro+UC – Sham+UC	Post	−0.55 ± 0.62 [−1.78, 0.67]	−0.892 [127], 0.374
	Chiro+UC – Sham+UC	Follow-up	−0.31 ± 0.7 [−1.7, 1.08]	−0.439 [105], 0.662

These results show no significant difference for any subscale score. A/D, Aggression/Defiance; CI, confidence intervals; HI, Hyperactivity and Impulsivity; IA, Inattention; ODD, Oppositional defiant disorder; SE, standard error.

#### 3.2.2 SNAP-IV

The within-group analysis of the SNAP-IV scale showed a significant reduction in the Aggression/Defiance, Hyperactivity and Impulsivity, Inattention, and Oppositional defiant disorder in both the Chiro+UC group and the Sham+UC group at four-week post-intervention and eight-week follow-up (Table 4). In the between-group analysis of SNAP-IV, there was no significant difference in the Aggression/Defiance, Hyperactivity and Impulsivity, Inattention, and Opposition/Defiance between the Chiro+UC and Sham+UC group at four-week post-intervention and eight-week follow-up (Table 5).

**Table 4:** Within-group change scores for the Swanson, Nolan and Pelham Teacher and Parents Rating Scale (SNAP-IV).

Outcome	Group	Time	Estimate ± SE [95% CI]	H_0_: Estimate = 0 t [df], *p*-value
Total Score	Chiro+UC	Post	0.8 ± 0.1[0.5, 1]	7.021 [95.7], <0.001
	Sham+UC	Post	0.8 ± 0.1[0.5, 1]	6.605 [95.7], <0.001
	Chiro+UC	Follow-up	0.7 ± 0.1[0.5, 0.9]	6.632 [95.7], <0.001
	Sham+UC	Follow-up	0.7 ± 0.1[0.5, 1]	6.069 [95.7], <0.001
A/D	Chiro+UC	Post	0.87 ± 0.13 [0.61, 1.13]	6.645 [85], <0.001
	Sham+UC	Post	1.02 ± 0.14 [0.74, 1.29]	7.35 [86], <0.001
	Chiro+UC	Follow-up	0.77 ± 0.12 [0.54, 1]	6.679 [93], <0.001
	Sham+UC	Follow-up	0.90 ± 0.13 [0.64, 1.16]	6.915 [96], <0.001
CI	Chiro+UC	Post	0.69 ± 0.13 [0.43, 0.95]	5.269 [85], <0.001
	Sham+UC	Post	0.65 ± 0.14 [0.37, 0.92]	4.7 [85], <0.001
	Chiro+UC	Follow-up	0.72 ± 0.12 [0.49, 0.95]	6.224 [94], <0.001
	Sham+UC	Follow-up	0.79 ± 0.13 [0.53, 1.05]	6.093 [95], <0.001
H/Im	Chiro+UC	Post	0.75 ± 0.13 [0.49, 1.01]	5.735[85], <0.001
	Sham+UC	Post	0.68 ± 0.14 [0.41, 0.95]	4.926 [85], <0.001
	Chiro+UC	Follow-up	0.67 ± 0.12 [0.44, 0.9]	5.787 [93], <0.001
	Sham+UC	Follow-up	0.49 ± 0.13 [0.23, 0.75]	3.79 [95], <0.001
I/O	Chiro+UC	Post	0.68 ± 0.13 [0.42, 0.94]	5.207 [85], <0.001
	Sham+UC	Post	0.55 ± 0.14 [0.27, 0.82]	3.942 [86], <0.001
	Chiro+UC	Follow-up	0.67 ± 0.12 [0.44, 0.9]	5.768 [93], <0.001
	Sham+UC	Follow-up	0.65 ± 0.13 [0.4, 0.91]	5.027 [95], <0.001
ADHD-In	Chiro+UC	Post	0.70 ± 0.13 [0.44, 0.96]	5.319 [85], <0.001
	Sham+UC	Post	0.56 ± 0.14 [0.29, 0.84]	4.08 [86], <0.001
	Chiro+UC	Follow-up	0.78 ± 0.12 [0.55, 1.01]	6.768 [94], <0.001
	Sham+UC	Follow-up	0.70 ± 0.13 [0.44, 0.96]	5.405[95], <0.001
OD	Chiro+UC	Post	0.92 ± 0.13 [0.66, 1.18]	7.018 [85], <0.001
	Sham+UC	Post	0.94 ± 0.14 [0.67, 1.22]	6.792 [87], <0.001
	Chiro+UC	Follow-up	0.72 ± 0.12 [0.49, 0.95]	6.187 [93], <0.001
	Sham+UC	Follow-up	0.87 ± 0.13 [0.61, 1.13]	6.634 [98], <0.001

ADHD-In, Attention Deficit Hyperactivity Disorder-Inattention; A/D, Aggression/Defiance; CI, Conners Index; H/Im, Hyperactivity and Impulsivity; I\O, Inattention\Overactivity; OD, Opposition/Defiance; SE, standard error.

**Table 5:** Between-group differences for the Swanson, Nolan and Pelham Teacher and Parents Rating Scale (SNAP-IV).

Outcome	Contrast	Time	Difference ± SE [95% CI]	H_0_: Difference = 0 t [df], *p*
Total Score	Chiro+UC – Sham+UC	Post	0 ± 0.2 [−0.3, 0.3]	0.036 [95.7], 0.971
	Chiro+UC – Sham+UC	Follow-up	0 ± 0.2 [−0.3, 0.3]	0.121 [97.3], 0.904
A/D	Chiro+UC – Sham+UC	Post	−0.15 ± 0.19 [−0.53, 0.23]	−0.778 [85], 0.439
	Chiro+UC – Sham+UC	Follow-up	−0.13 ± 0.17 [−0.47, 0.22]	−0.739 [95], 0.462
C/I	Chiro+UC – Sham+UC	Post	0.04 ± 0.19 [−0.34, 0.42]	0.212 [85], 0.832
	Chiro+UC – Sham+UC	Follow-up	−0.07 ± 0.17 [−0.42, 0.28]	−0.401 [94], 0.689
H/Im	Chiro+UC – Sham+UC	Post	0.07 ± 0.19 [−0.31, 0.45]	0.366 [85], 0.715
	Chiro+UC – Sham+UC	Follow-up	0.18 ± 0.17 [−0.17, 0.52]	1.021 [94], 0.310
I/O	Chiro+UC – Sham+UC	Post	0.14 ± 0.19 [−0.24, 0.51]	0.712 [85], 0.478
	Chiro+UC – Sham+UC	Follow-up	0.01 ± 0.17 [−0.33, 0.36]	0.082 [94], 0.935
ADHD-In	Chiro+UC – Sham+UC	Post	0.13 ± 0.19 [−0.25, 0.51]	0.69 [85], 0.492
	Chiro+UC – Sham+UC	Follow-up	0.08 ± 0.17 [−0.26, 0.43]	0.467 [94], 0.642
OD	Chiro+UC – Sham+UC	Post	−0.03 ± 0.19 [−0.41, 0.35]	−0.138 [86], 0.891
	Chiro+UC – Sham+UC	Follow-up	−0.16 ± 0.17 [−0.5, 0.19]	−0.887 [96], 0.377

These results show no significant between-group differences across any subscale score. ADHD-In, Attention Deficit Hyperactivity Disorder-Inattention; A/D, Aggression/Defiance; CI, Conners Index; H/Im, Hyperactivity and Impulsivity; I\O, Inattention\Overactivity; OD, Opposition/Defiance; SE, standard error.

#### 3.2.3 ADHD Rating Scale-IV

In the within-group analysis of the ADHD Rating Scale-IV, the total score, hyperactivity and impulsivity subscale scores significantly decreased in the Chiro+UC group at four-weeks’ post-intervention and eight-weeks’ follow-up, while there was no significant change at either timepoint in the Sham+UC group. The inattention subscale significantly decreased within both the Chiro+UC and Sham+UC groups at four-week post-intervention and at eight-weeks’ follow-up (Table 6). The between-group analysis of ADHD Rating Scale-IV showed there was no significant difference in the total score, hyperactivity and impulsivity and inattention between the Chiro+UC group and Sham+UC group at four-weeks’ post-intervention and eight-weeks’ follow-up (Table 7).

**Table 6:** Within-group change-scores for the Attention-Deficit/Hyperactivity Disorder (ADHD) Rating Scale-IV.

Outcome	Group	Time	Estimate ± SE [95% CI]	H_0_: Estimate = 0 t [df], *p*-value
Total Score	Chiro+UC	Post	11 ± 2 [7, 15]	5.227 [92.6], <0.001
	Sham+UC	Post	10 ± 2 [6, 15]	4.738 [92.3], <0.001
	Chiro+UC	Follow-up	11 ± 2 [7, 15]	5.16 [92.6], <0.001
	Sham+UC	Follow-up	10 ± 2 [5, 14]	4.304 [95.5], <0.001
HI	Chiro+UC	Post	10 ± 4 [2, 19]	2.37 [87], 0.020
	Sham+UC	Post	9 ± 5 [−1, 18]	1.857 [87], 0.067
	Chiro+UC	Follow-up	10 ± 5 [1, 20]	2.159 [75], 0.034
	Sham+UC	Follow-up	8 ± 5 [−3, 19]	1.497 [77], 0.138
IA	Chiro+UC	Post	21 ± 4 [13, 30]	4.852 [87], <0.001
	Sham+UC	Post	11 ± 5 [2, 20]	2.408 [87], 0.018
	Chiro+UC	Follow-up	21 ± 5 [12, 31]	4.429 [74], <0.001
	Sham+UC	Follow-up	21 ± 5 [11, 32]	3.923 [77], <0.001

CI, confidence intervals; HI, Hyperactivity and Impulsivity; IA, Inattention; SE, standard error.

**Table 7:** Between-group differences.

Outcome	Contrast	Time	Difference ± SE [95% CI]	H_0_: Difference = 0 t [df], *p*-value
Total Score	Chiro+UC – Sham+UC	Post	0 ± 3 [−6, 6]	0.135 [92.1], 0.893
	Chiro+UC – Sham+UC	Follow-up	1 ± 3 [−5, 7]	0.233 [93.9], 0.816
HI	Chiro+UC – Sham+UC	Post	2 ± 6 [−11, 15]	0.281 [87], 0.779
	Chiro+UC – Sham+UC	Follow-up	2 ± 7 [−12, 17]	0.299 [76], 0.765
IA	Chiro+UC – Sham+UC	Post	10 ± 6 [−3, 23]	1.557 [88], 0.123
	Chiro+UC – Sham+UC	Follow-up	0 ± 7 [−15, 14]	−0.045 [77], 0.964

CI, confidence intervals; HI, Hyperactivity and Impulsivity; IA, Inattention; SE, standard error.

## 4 Discussion

This pilot randomized controlled trial was the first to investigate the feasibility and efficacy of 4 weeks of chiropractic care on inattention, hyperactivity, and impulsivity in children with ADHD. The experimental design in this population was feasible, with satisfactory recruitment, blinding, data collection, follow-up, and safety. The between-group analyses in this study did not show significant differences between the intervention and control group at any assessment time point in any total or subscale ADHD outcome measure. However, it is important to interpret these findings with caution, as this was a pilot study with a relatively small sample size.

Both groups showed significant within-group differences on the VADTRS and SNAP-IV scales post-intervention, which can be explained by the study design including usual standard-of-care interventions for both cohorts (e.g., cognitive, speech, and physical therapy). These within-group improvements were retained post-intervention and were statistically significant at follow up for the VADTRS and SNAP-IV scale apart from inattention in the sham group, which was insignificant (*p* = 0.067). There were substantial improvements within the chiropractic group on the ADHD Rating Scale-IV in hyperactivity/impulsivity post-intervention which were retained until the follow-up session, compared to the sham group which showed no significant improvements. Significant improvements were seen mainly in hyperactivity/impulsivity and inattention, signifying improvements in behavioral symptoms of ADHD. However, it is important to note that these results were not statistically significant, which may be due to the small sample size. A larger scale study would be needed to further investigate these results. Given the preliminary nature of these results, caution is advised in interpreting their clinical implications. The present pilot study was not adequately powered to test hypotheses regarding the clinical benefit of chiropractic for ADHD; rather, it aimed to assess the feasibility of the experimental design. While our findings do not definitively rule out the potential benefit of chiropractic adjustment in certain patients with ADHD, particularly those with conditions known to respond positively to this treatment, such as spinal pain, chiropractic clinicians may consider co-managing patients with ADHD as part of a multidisciplinary team, on a case-by-case basis, depending on the clinical context and adherence to best practice guidelines (Keating et al., 2023).

Several markers of feasibility testing were favorable in the present study. The duration of patient recruitment only spanned 2 weeks, suggesting a larger sample size could be met in a follow-up study with a longer enrolment window. The retention rate of the present study (84%) can be considered high considering the need for multiple treatment sessions and assessment periods and dropouts due to school absences. However, it is difficult to evaluate the success of the retention rate as we are unaware of similar trials including children with ADHD receiving a chiropractic intervention. Although this pilot trial was of a small size, it adds to the limited existing literature supporting the safety of chiropractic adjustments in the pediatric population (Swait and Finch, 2017; Chu et al., 2023). The follow-up assessment demonstrated excellent participant retention, with no dropouts reported within the assessment window, highlighting the feasibility and commitment of the study participants. Furthermore, the study’s success in blinding is noteworthy, as over 95% of teachers across both intervention groups perceived the children to have undergone an active chiropractic intervention, irrespective of their actual group allocation. Achieving such high blinding success in a manual intervention trial is a significant strength, enhancing the internal validity of the study and mitigating potential bias in participant and teacher perceptions. Therefore, the present study suggests that it is feasible to examine the efficacy of chiropractic adjustment in children with ADHD. Researchers may consider building upon our study and refining the methods further as desired based on the limitations (see below).

The qualitative feedback from the teachers indicated improvement in the childrens’ behavior suggesting that the chiropractic adjustments may have had an impact on reducing inattention, hyperactivity, and impulsivity in the children. However, there were no significant between-group differences in our statistical analysis. This discrepancy between qualitative and quantitative findings may imply that the chosen outcome measures were not optimal to capture the effects of the intervention. The outcome measures utilized in the study (i.e., VADTRS, SNAP-IV, and ADHD Rating Scale IV) incorporated ratings from teachers and parents, a strategy recommended to reflect changes in ADHD symptoms both within and outside of the classroom (Collett et al., 2003; Pelham et al., 2005; Pliszka and AACAP Work Group on Quality Issues, 2007; Epstein and Weiss, 2012). However, these study measures remain subjective and therefore may have introduced bias. Subjectivity in ADHD scoring measures may result from inter-rater differences (e.g., discrepancies between teachers or teacher-parent scores) and contextual factors (i.e., related to the within-school setting) (Collett et al., 2003). As a result, these scales may not provide a purely objective representation of the changes in ADHD symptoms. To address this limitation and improve future trials, we recommend incorporating objective measures of ADHD symptoms. These could include neuropsychological assessments (Emser et al., 2018) or even neuroimaging techniques, that provide more direct and quantifiable measurements. By employing such measures, researchers can ensure a more valid evaluation of the effects of chiropractic adjustment on ADHD symptoms.

The preliminary null results of this pilot trial can be contrastedwith previous case reports that noted a beneficial response to chiropractic adjustment amongst children with ADHD. While it may be challenging to reconcile the seemingly conflicting results to the present study, it should be noted that case reports are uncontrolled observational studies and tend to highlight unique clinical scenarios (Wittman et al., 2009; Alcantara and Davis, 2010; Muir, 2012; Fairest et al., 2019). In addition, in two such case reports, the child also had back pain or recent fall on the head suggesting that treatment of spinal pain may have accounted for some symptom benefit (Muir, 2012; Fairest et al., 2019). In contrast, spinal pain or injury were not required inclusion criteria in the current study. While there is limited epidemiologic information on this topic, somatic complaints such as spinal pain or headache are commonly described comorbidities accompanying ADHD in children (Leirbakk et al., 2015). Accordingly, lack of requirement of comorbid somatic or spinal symptoms may have influenced the findings of the present study. It remains unclear if the presence of such symptoms would have led to different results.

The results from the present study may also be compared with research regarding the effect of chiropractic adjustments on oculomotor control. Studies have identified a role of frontal eye fields in the regulation of attention (Funahashi and Andreau, 2013). One recent study found that chiropractic adjustment led to improved oculomotor control and reading time in children with ADHD (Cade et al., 2021). The study’s authors proposed that as oculomotor control is thought to rely on accurate sensory processing, the changes in the afferent input after spinal adjustment could have accounted for the observed positive treatment response. However, In the present study, there were no significant between-group differences in attention, and oculomotor markers and reading time were not directly measured, thus neither corroborating nor refuting the previous study’s findings.

### 4.1 Strengths and limitations

Statistically significant between-group improvements were not observed in any ADHD outcome measure at four and 8 weeks with a final sample of 56 participants. This suggests that this sample size may not have been large enough to detect between-group changes in the outcomes measured. As a pilot study, we utilized a variety of outcome measures and made multiple comparisons without making adjustments to *p*-values. While this strategy increases the chances of making type I errors, it is considered to be appropriate when exploring new areas of research such as this (Feise, 2002). Future large scale RCTs using similar outcome measures can therefore use the estimates of this study to facilitate *a priori* sample size calculations. In addition, future research should utilize longer intervention and follow-up periods which may be needed to observe long-term between-group differences in this population. Baseline or past use of ADHD medications was unavailable in the school records, therefore we were unable to report on this variable. We did not record any potential disagreements between teachers and parents in scoring of the ADHD assessment measures, which required collaborative input from both parties. As these scales have some subjectivity (Collett et al., 2003), we encourage future trials to incorporate more objective outcome measures to diminish this potential bias (Emser et al., 2018). It is possible that an observer bias accounted for improvements in ADHD measures within both groups. Despite blinding, teachers were aware that students were participating in the study and therefore may have desired to see positive changes, thereby unconsciously reporting improvements across the ADHD outcome measures in both groups (Steiner et al., 2013). The methods of this study relied on partnering with a specialized multidisciplinary school and therefore a similar study may not be as feasible in regions that do not have access to such a setting.

## 5 Conclusion

Our experimental design examining chiropractic care for children with ADHD was feasible in terms of recruitment, retention, data collection, randomization and blinding, qualitative feedback, safety, and adherence. There were no significant between-group improvements in ADHD outcome measures when chiropractic spinal adjustment was added to 4 weeks of usual care. Given the significance of within-group changes, limited sample size, and use of a standard-of-care intervention in both groups, efficacy of chiropractic adjustments for ADHD symptoms cannot be ruled out with the present study design. Further research, involving larger group sizes, longer-term follow-up and intervention periods, and more objective outcome measures is required to more definitively investigate the effects of chiropractic spinal adjustments on cognitive and executive functions in children with ADHD.

## Data availability statement

The datasets presented in this article are not readily available because it can potentially be shared, but approval from the Ethics board would be requested, and the requester needs to follow the procedure at the time of the request. Requests to access the datasets should be directed to imran.niazi@nzchiro.co.nz.

## Ethics statement

The studies involving humans were approved by Ethical Review Committee of Riphah International University, Pakistan (Riphah/RCRS/REC/000459). The studies were conducted in accordance with the local legislation and institutional requirements. Written informed consent for participation in this study was provided by the participants' legal guardians/next of kin. Written informed consent was obtained from the individual(s) for the publication of any identifiable images or data included in this article.

## Author contributions

IA: Conceptualization, Data curation, Formal analysis, Investigation, Methodology, Software, Visualization, Writing – original draft, Writing – review & editing. IN: Conceptualization, Funding acquisition, Investigation, Methodology, Project administration, Resources, Software, Supervision, Validation, Visualization, Writing – original draft, Writing – review & editing. NK: Conceptualization, Data curation, Investigation, Methodology, Validation, Visualization, Writing – review & editing. JensD: Investigation, Methodology, Project administration, Resources, Supervision, Writing – review & editing. GS: Data curation, Investigation, Methodology, Validation, Visualization, Writing – review & editing. UR: Formal analysis, Investigation, Methodology, Software, Visualization, Writing – review & editing. JennD: Investigation, Methodology, Validation, Visualization, Writing – review & editing. RT: Investigation, Methodology, Validation, Visualization, Writing – review & editing. KH: Conceptualization, Funding acquisition, Investigation, Methodology, Project administration, Resources, Supervision, Writing – review & editing. HH: Conceptualization, Funding acquisition, Investigation, Methodology, Project administration, Resources, Supervision, Validation, Visualization, Writing – review & editing.
